# Hyperinsulinemia impairs the metabolic switch to ketone body utilization in proximal renal tubular epithelial cells under energy crisis *via* the inhibition of the SIRT3/SMCT1 pathway

**DOI:** 10.3389/fendo.2022.960835

**Published:** 2022-09-27

**Authors:** Jinlan Xie, Feifei Zhong, Zhenhong Guo, Xinran Li, Jingyu Wang, Zhongai Gao, Baocheng Chang, Juhong Yang

**Affiliations:** Department of Endocrinology, National Health Commission Key Laboratory of Hormones and Development Tianjin Key Laboratory of Metabolic Diseases, Endocrinology Institute of Chu Hsien-I Memorial Hospital and Tianjin Institute of Endocrinology, Tianjin Medical University, Tianjin, China

**Keywords:** Hyperinsulinemia, metabolic flexibility, proximal tubular epithelial cells, mitochondria, β-hydroxybutyrate

## Abstract

**Objective:**

To investigate the effects and mechanism of hyperinsulinemia on the metabolic switch to β‐hydroxybutyrate (BHB) absorption and utilization under a starvation or hypoxic environment in proximal tubular epithelial cells.

**Methods:**

A high-fat diet-induced hyperinsulinemia model in ZDF rats was used to test the expression of key enzymes/proteins of ketone body metabolism in the kidney. Notably, 12-week-old renal tubule SMCT1 specific knockout mice (SMCT1 flox/floxCre+) and control mice (SMCT1 flox/floxCre-) were used to confirm the roles of SMCT1 in kidney protection under starvation. The changes of key enzymes/proteins of energy metabolism, mitochondrial function, and albumin endocytosis in HK2 cells under low glucose/hypoxic environments with or without 50 ng/mL insulin were studied. Silent information regulation 2 homolog 3 (SIRT3) was overexpressed to evaluate the effect of hyperinsulinemia on the metabolic switch to BHB absorption and utilization through the SIRT3/SMCT1 pathway in HK2 cells.

**Results:**

In ZDF rats, the expression of HMGCS2 increased, the SMCT1 expression decreased, while SCOT remained unchanged. In renal tubule SMCT1 gene-specific knockout mice, starvation for 48 h induced an increase in the levels of urine retinol-binding protein, N-acetyl-β-glucosaminidase, and transferrin, which reflected tubular damages. In HK2 cells under an environment of starvation and hypoxia, the levels of key enzymes related to fatty acid oxidation and ketone body metabolism were increased, whereas glucose glycolysis did not change. The addition of 2 mmol/l BHB improved ATP production, mitochondrial biosynthesis, and endocytic albumin function, while cell apoptosis was reduced in HK2 cells. The addition of 50 ng/ml insulin resulted in the decreased expression of SMCT1 along with an impaired mitochondrial function, decreased ATP production, and increased apoptosis. The overexpression of SIRT3 or SMCT1 reversed these alterations induced by a high level of insulin both in low-glucose and hypoxic environments.

**Conclusions:**

The increased absorption and utilization of BHB is part of the metabolic flexibility of renal tubular epithelial cells under starvation and hypoxic environments, which exhibits a protective effect on renal tubular epithelial cells by improving the mitochondrial function and cell survival. Moreover, hyperinsulinemia inhibits the absorption of BHB through the inhibition of the SIRT3/SMCT1 pathway.

## 1 Background

Metabolic flexibility (MF) is the ability of an organism to adapt to substrate oxidation rates in response to the changes in fuel availability (1), which depends on the metabolic pathways of tissue management, sensing, uptake, transport, storage, and utilization of energy substrates. The synthesis, degradation, and activity of key proteins and enzymes in the metabolic circuit play an important role in the regulation of MF.

Physiologically, the proximal tubule preferentially uses fatty acid β oxidation for energy supply, whereas ketone bodies have very little function. Yet during starvation, the renal reabsorption of ketones can increase to fully cover the energy needs of the kidneys (2). In addition, it has been shown that in a hypoxic environment as in acute kidney injury (AKI), the genes related to ketone body metabolism and fatty acid oxidation are upregulated, which, in turn, resist multiple cellular damages (3). Moreover, an increasing number of studies have shown that β‐hydroxybutyrate (BHB) plays a role in anti-oxidative stress, anti-inflammatory mechanisms, and mitochondrial function protection (4). Mitochondria play an important role in MF and the maintenance of mitochondrial function contributes to normal cellular energy metabolism, whereas an impaired mitochondrial function leads to reduced MF as observed in patients with diabetic nephropathy (5). However, mechanisms leading to impaired MF in diabetic nephropathy are not clear, especially regarding the metabolism of ketone bodies.

Insulin resistance and its concomitant hyperinsulinemia are abnormal physiological states and common risk factors for several diseases, such as hypertension, diabetes, obesity, and atherosclerosis, which can cause various renal complications (6, 7). Our previous study (8) found that 12.1% of patients with impaired glucose tolerance suffered from microalbuminuria; these patients exhibited reabsorption dysfunction, while 58.3% of patients had tubular structural damage. Moreover, our study found that hyperinsulinemia may mediate these injuries to renal tubules, while the underlying mechanism remains unclear. Yi et al. (9) found that the BHB level in renal tissue was decreased significantly during the period of hyperinsulinemia despite the high circulation level of BHB in obese rats. Therefore, we speculate that hyperinsulinemia may impair the MF by decreasing the absorption and utilization of BHB in the kidney during hypoxic or low-glucose situations and cause kidney damages because of energy crisis. In this study, we first used a high-fat diet-induced hyperinsulinemia model to test the expression of key enzymes/proteins involved in ketone body metabolism in the kidney. Subsequently, we used renal tubule SMCT1 gene-specific knockout mice to explore whether reduced BHB resorption is involved in renal tubular injury under starvation. We investigated the metabolic switch to BHB using HK2 cells and its impact on the mitochondrial function or endocytosis under a low-glucose, hypoxic environment with or without high level of insulin. We also explored the pathway through which high insulin may function as an inhibitor of the BHB switch during an energy crisis.

## 2 Experimental subjects and methods

### 2.1 Experimental subjects

Ten 6-week-old male ZDF rats and 10 control ZL rats were purchased from Beijing Weitonglihua Experimental Animal Technology Co., Ltd.

Four 8-week-old female SMCT1flox and two 8-week-old male Cdh16-cre mice (F1 generation) were purchased from Shanghai Nanfang Model Biotechnology Co., Ltd. for breeding. Subsequently, 12 mice with renal tubule SMCT1 gene-specific knockout at the age of 12 weeks (SMCT1 flox/floxCre+) and 12 control mice (SMCT1 flox/floxCre-) were used to study the roles of SMCT1 in tubular protection under energy stress.

Human renal cortical proximal tubular epithelial cells (HK2 cells) were purchased from the cell bank of the Chinese Academy of Sciences.

### 2.2 Experimental methods

#### 2.2.1 Animals

##### 2.2.1.1 ZDF diabetic rats

Twenty 8-week-old male ZDF rats were fed adaptively with normal chow for 1 week in a clean and well-ventilated environment with water and bedding changed once every 3 days. Subsequently, 10 rats were maintained as a healthy control group and were fed normal chow, while the others were fed high-fat chow. An OGTT test was performed every week. The diabetes modeling was a success if the peak blood glucose post-glucose load was greater than 16.0 mmol/l or the 2-h blood glucose post-glucose load was greater than 11.1 mmol/l. The blood, urine samples, and kidney specimens were collected and stored at –80̊ for further analysis.

##### 2.2.1.2 Renal tubule SMCT1 gene-specific knockout mice

The SMCT1flox mice (referred to as SMCT1 conditional knockout, SMCT1 CKO) were produced using the CRISPR/Cas9 system. Renal tubule SMCT1 gene-specific knockout mice were generated using the Cre-loxP system by crossing Cdh16-cre mice with SMCT1flox mice. The offspring with a genotype of positive Cdh16-cre transgene and SMCT1^flox/+^ were called heterozygous mice with two genes, which were reserved and selected. The double-positive mice were subsequently mated with SMCT1 CKO mice in the second round of hybridization, and the offspring with a genotype of positive Cdh16-cre transgene and SMCT1flox/flox were selected as renal tubule SMCT1 gene-specific knockout (SMCT1 ^flox/floxCre+^) mice and the corresponding control group was SMCT1 flox/floxCre-. Twelve mice, placed in two cages of six each, were fed during the experiments, and the feed and water were changed every 3 d. The mice were fed an ordinary diet. All the mice were housed in an SPF animal room with the temperature and humidity maintained at 20°C–25°C and 50%–70%, respectively. The mice were fasted overnight for blood collection to measure the plasma biochemical data between the two groups. We also collected blood and urine samples after the mice showed hunger for 48 h at 12 weeks of age.

##### 2.2.1.3 General information and biochemical markers

Biochemical indicators were detected using a Roche C701 automatic biochemical analyzer. UMA was measured by immunoturbidimetry; retinol-binding protein (RBP) was measured by an ELISA kit, N-acetyl-β-glucosaminidase (NAG) was measured by an MNP-G1CNAC substrate method, and the sodium+ (Na+) content was measured by a colorimetric method. All the specimens were tested in the Department of Clinical Laboratory at Tianjin Medical University Chu Hsien-I Memorial Hospital and Tianjin YiShengYuan Bio-Technology Co. Ltd.

#### 2.2.2 HK2 cells and interventions

HK2 cells were cultured in a medium containing 90% DMEM/F12 medium (SH30023.01, HyClone, USA), 10% fetal bovine serum (FBS, 0500, Sciencell, USA), and 1% penicillin/streptomycin (P1400, Solarbio Beijing, China) in an incubator at 37°C and 5% CO2. As the glucose concentration in the DMEM/F-12 medium was 17.49 mmol/l (normal control, NC group), and the minimum glucose concentration for HK2 cell to survive was 5.5 mmol/l, the glucose concentration of 5.5 mmol/l was used as a starvation environment (low glucose, LG group) in our study. Cells in the hypoxic group were incubated in a hypoxic three-gas incubator (5% CO2, 1% O2, 94% N2, Hypoxic group). The BHB (CAS:150-83-4, Sigma, USA) intervention concentration was 2 mmol/l. Subsequently, according to our previous study (8), we used 5 ng/ml insulin as the physiological concentration and 50 ng/ml as the high concentration. All the above interventions were conducted for 24 h.

#### 2.2.3 Western blot

A Western blot was carried out to detect the expression of carnitine palmityl transferase 1 (CPT1α, ab234111, Abcam, UK), pyruvate kinase isozymes M2 (PKM2, 4053S-100UL, Cell Signaling Technology, CST, USA), 3-hydroxy-3-methylglutaryl- Coenzyme A synthase 2 (HMGCS2, ab137043, Abcam, UK), Na+-coupled monocarboxylate transporter (SMCT1, orb101289, Biorbyt, UK), succinyl-CoA:3-ketoacid CoA transferase (SCOT, ab224250, Abcam, UK), peroxisome proliferator activated receptor co-activator-1α (PGC1α, NBP1-04676, Novus, USA) (PGC1α, ab191838, Abcam, UK), mitochondrial fusion protein 1 (Mfn1, 14739S, Cell Signaling Technology, CST, USA), mitochondrial dynamin-related protein 1 (Drp1, NB110-55288, Novus, USA), Bcl-2 (A19693, Abclonal, Wuhan, China), Bax (A12009, Abclonal, Wuhan, China), and silent information regulation 2 homolog 3 (SIRT3, 2627S, Cell Signaling Technology, CST, USA) proteins. Among them, CPT1α, PKM2, HMGCS2, SMCT1, SCOT, PGC1α, Mfn1, Drp1, Bcl2, Bax, and SIRT3 were all rabbit polyclonal antibodies with a dilution of 1:1000, while GAPDH was a mouse monoclonal antibody with a dilution of 1:3000.

#### 2.2.4 RNA extraction and real-time PCR

Trizol Reagent (15596026, Invitrogen, USA) was used to extract RNA from the treated cells. cDNA was synthesized using the reverse transcription system kit (K1622, Thermo, USA). Additionally, qRT-PCR was performed using the Applied Biosystems 2700 *via* Direct SYBR Green PCR kit (Sangon Biotech, Shanghai, China). PCR amplification was carried out under the following conditions: DNA was denatured at 94°C for 5 min followed by 35 cycles of denaturation at 94°C for 30 s, annealing at 55°C for 30 s, and extension at 72°C for 30 s. The following primer sequences were used:

PGC1α forward: 5′-GTCACCACCCAAATCCTTAT-3′,
reverse: 5′-ATCTACTGCCTGGAGACCT-3′;
Mfn1 forward: 5′-GTGGCAAACAAAGTTTCATGTG-3′,
reverse: 5′-CACTAAGGCGTTTACTTCATCG-3′;
Drp1 forward: 5′-AATGCAGCCAAGATCTTTAACC-3′,
reverse: 5′-TTGTGACTTTTGGCTGTTATGG-3′;
Bcl2 forward: 5′-GACTTCGCCGAGATGTCCAG-3′,
reverse: 5′-GAACTCAAAGAAGGCCACAATC-3′;
Bax forward: 5′-CGAACTGGACAGTAACATGGAG-3′,
reverse: 5′-CAGTTTGCTGGCAAAGTAGAAA-3′;
SMCT1 forward: 5′-CCAGCAGACCTCCAAGGACT-3′,
reverse: 5′-GGACAGTGACGGCTGACATG-3′;
SIRT3 forward: 5′-GCCATTTTTGAACTCCCATTCT-3′,
reverse: 5′-GGAGAAAGTAGTGAGTGACGTT-3′.

#### 2.2.5 ATP production

The ATP production was measured according to the kit (S0026, Beyotime, Shanghai, China) instructions; the RLU value was measured and calculated according to the standard curve to obtain the ATP concentration of the sample using a Luminometer (Biotek, Germany). The protein concentration in the sample was determined using the BCA Protein Concentration Assay Kit (PC0020-500, Solarbio Beijing, China). The concentration of ATP was subsequently converted to the form of nmol/mg protein.

#### 2.2.6 Reactive oxygen species

A reactive oxygen species (ROS) Assay Kit (CA1410, Solarbio Beijing, China) was used to detect the cellular ROS production. Adherent cells cultured in 24-well plates were added to 250 μL of DCFH-DA at a concentration of 10 μmol/l and incubated for 20 min at 37°C in a cell incubator. Subsequently, the cells were washed with a serum-free cell culture medium thrice and finally incubated with a normal culture medium for direct observation by fluorescence microscopy (Olympus Corp., Tokyo, Japan).

#### 2.2.7 Albumin endocytosis using FITC-BSA

The cells were cultured in 96-well or 24-well plates and were then stimulated with 500 μg/ml FITC-BSA (SF063, Solarbio Beijing, China) for 4 h in a dark place. Subsequently, the cells were washed with PBS twice. The intensity of fluorescence released from the cells, reflecting the cellular reabsorption function, in 96-well plates was measured using an automatic microplate reader at 493 nm excitation wavelength and 550 nm emission wavelength. The cells in 24-well plates were directly observed using a fluorescence microscope (Olympus Corp., Tokyo, Japan).

#### 2.2.8 Cell membrane potential

The cell membrane potential was measured using a mitochondrial membrane potential assay kit (Beijing Solarbio Science & Technology Co., Ltd) with JC-1. First, the staining working solution was prepared according to the instructions. Subsequently, cells cultured in the 24-well plates were added with 250 μL of JC-1 staining working solution and mixed thoroughly. Next, the cells were incubated at 37°C for 20 min in the cell incubator; the supernatant was aspirated and washed twice with JC-1 staining buffer (1X). Finally, the cells were added to a 250 μL cell culture medium and observed under a fluorescence microscope (Olympus Corp., Tokyo, Japan).

#### 2.2.9 Mitochondria number

A MitoTracker® Red CMXRos Mitochondrial Red Fluorescent Probe (M9940, Solarbio Beijing, China) was used to detect the number of mitochondria. The staining working solution was prepared in advance according to the kit instructions. After staining, the cells were refreshed with cell culture solution or buffer and were read at Ex = 579 nm and Em = 599 nm.

#### 2.2.10 Intracellular BHB levels

Approximately 200 μL of lysate was added to the six-well plate to lysate the cells. After full lysis, centrifugation was performed at 4̊ and 1500×g for 10 min, and the supernatant was collected for subsequent determination. A human β-hydroxybutyrate ELISA Kit (10290, Lunchangshuo Biotechnology Co., Ltd., Xiamen, China) was rewarmed for 30 min; the instructions of the kit were followed, and a microplate reader was used for the reading. A wavelength of 450 nm was used to measure the optical density value (OD value) of each well. The values were calculated according to the standard curve and compared with BCA.

#### 2.2.11 Transfection of overexpressed reagents

SMCT1 overexpression (AAV-SMCT1) and homologous negative control (AAV-SMCT1 NC), SIRT3 overexpression (pEX-1-SIRT3), and homologous negative control [pcDNA3.1(+)] were all purchased from GenePharma (Suzhou, China). According to the manufacturer’s instructions, the cells were transfected using lipofectamine 3000 reagent (Invitrogen, CA, USA) at a final concentration of 50–100 mmol/l.

### 2.3 Statistical analysis

The data were counted and plotted using the Graphpad software. The measurement data were tested for normality. The data that conformed to a normal or approximately normal distribution were expressed as “mean ± standard deviation (x ± s)”, while those that did not conform to a normal distribution were expressed as median. The difference between the two groups were compared by a t-test and the difference between multiple groups were compared by a one-way analysis of variance (ANOVA). The differences were considered as statistically significant at p < 0.05.

## 3 Results

### 3.1 Expression of the key enzymes of energy metabolism in the kidney of type 2 diabetic rats

Compared with the NC group, rats in the DM group had an increased level of blood sugar, ALT, TC, TG, insulin, and urine protein (p < 0.05) but were observed to have a decreased level of plasma SCr (p < 0.05). The body weight, AST, BUN, UA, and LDL levels were not significantly changed (p > 0.05) (**Table 1**).

**Table 1:** Comparison of general, blood, and urine biochemical data between the two groups of ZDF rats.

	NC	DM	p	*t*
Blood sugar (mmol/l)	4.36 ± 0.31	20.78 ± 0.96^a^	0.00	-36.42
Weight (g)	304 ± 11.62	286.75 ± 35.17	0.33	1.04
ALT (U/l)	58.62 ± 9.83	88.2 ± 19.91^a^	0.02	-2.94
AST (U/l)	165.74 ± 46.35	152.7 ± 64.67	0.73	0.35
BUN (mmol/l)	7.9 ± 0.45	9.06 ± 1.92	0.32	-1.17
SCr (µmol/l)	34.3 ± 3.11	21.8 ± 1.2^a^	0.00	8.24
UA (µmol/l)	60.06 ± 16.1	85.3 ± 23.85	0.10	-1.90
TC (mmol/l)	3.18 ± 0.21	4.34 ± 0.68^a^	0.01	-3.69
TG (mmol/l)	0.5 ± 0.08	3.73 ± 1.95^a^	0.045	-3.31
LDL (mmol/l)	2.06 ± 0.16	1.87 ± 1.01	0.73	0.37
Insulin	6.23 ± 0.74	10.82 ± 1.00^a^	0.00	-7.96
Urine protein (g/24 h)	0.13 ± 0.07	0.36 ± 0.05^a^	0.001	-5.52

ALT, alanine aminotransferase; AST, aspartate transaminase; BUN, blood urea nitrogen; SCr, serum creatinine; UA, uric acid; TC, total cholesterol; TG, triglyceride; LDL, low-density lipoprotein cholesterol. ^a^Compared with the NC group, p < 0.05.

**Figure 1:**
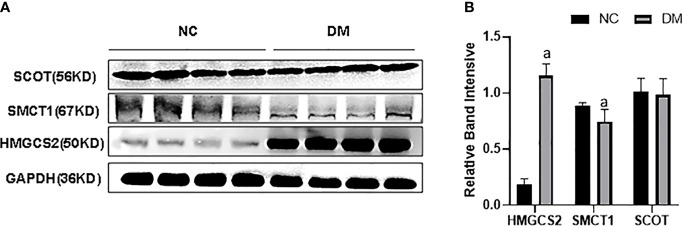
**(A, B)** Expression of HMGCS2, SMCT1, and SCOT in the renal cortex of type 2 diabetic rats.

We checked the expression of HMGCS2 reflecting ketone body synthase, SMCT1 reflecting ketone body reabsorption, and SCOT reflecting the utilization of ketone bodies in high-fat diet-induced type 2 diabetes. We found that the expression of HMGCS2 was increased, the expression of SMCT1 was decreased, while the expression of SCOT remained unchanged in the renal cortex of type 2 diabetic rats (**Figures 1A, B**).

### 3.2 Roles of SMCT1 in kidney protection in mice staved for 48 h

Mice in the SMCT1flox/flox Cre+ group showed a decreased plasma level of LA and BHB; however, similar levels of blood glucose, body weight, water intake, TG, TC, FFA, LDL, BUN, SCr, ALT, AST, and Na+ were observed compared to the control. Analysis of the blood samples from mice that were starved for 48 h revealed no significant differences in plasma BHB between the two groups **Table 2**.

**Table 2:** Baseline general data and plasma biochemical data between the two groups.

	SMCT1 ^flox/floxCre-^	SMCT1 ^flox/floxCre+^	p	*t*
Blood sugar (mmol/l)	9.44 ± 1.23	10.32 ± 1.71	0.16	1.44
Weight (g)	20.05 ± 3.34	19.83 ± 3.46	0.88	-0.16
Water intake (mL)	6.50 ± 1.68	6.25 ± 1.5	0.83	-0.22
TG (mmol/l)	1 ± 0.17	1.16 ± 0.24	0.33	1.03
TC (mmol/l)	2.82 ± 0.36	3.01 ± 0.29	0.39	0.91
FFA (mmol/l)	1.06 ± 0.15	1.38 ± 0.24	0.07	2.08
LDL-C (mmol/l)	0.12 ± 0	0.21 ± 0.14	0.14	1.72
BUN (mmol/l)	15.76 ± 3.4	13.21 ± 1.95	0.16	-1.54
SCr (µmol/l)	27.7 ± 1.35	27.21 ± 2.39	0.75	-0.32
UA (µmol/l)	143.3 ± 6.52	86.22 ± 9.08 ^a^	0.00	-9.40
ALT (U/l)	55.9 ± 11.13	46.54 ± 10.32	0.23	-1.29
AST (U/l)	152 ± 43.94	133.08 ± 37.47	0.54	-0.65
Na^+^ (mmol/l)	137.04 ± 11.46	134.51 ± 6.20	0.65	-0.47
BHB (mmol/l)	1.38 ± 0.53	0.64 ± 0.24 ^a^	0.03	-2.76
LA (mmol/l)	12.59 ± 0.29	13.44 ± 2.16	0.53	0.66
BHB (mmol/l)(Hunger for 48 h)	2.14 ± 0.52	2.08 ± 0.56	0.86	0.18

TG, triglyceride; TC, total cholesterol; FFA, free fatty acid; LDL-C, low-density lipoprotein cholesterol; BUN, blood urea nitrogen; SCr, Serum creatinine; UA, Uric acid; ALT, alanine aminotransferase; AST, aspartate transaminase; Na^+^, Sodium^+^; BHB, β-hydroxybutyrate; LA, Lactic acid. ^a^Compared with SMCT1^flox/flox cre-^ group, p < 0.05.

**Figure 2:**
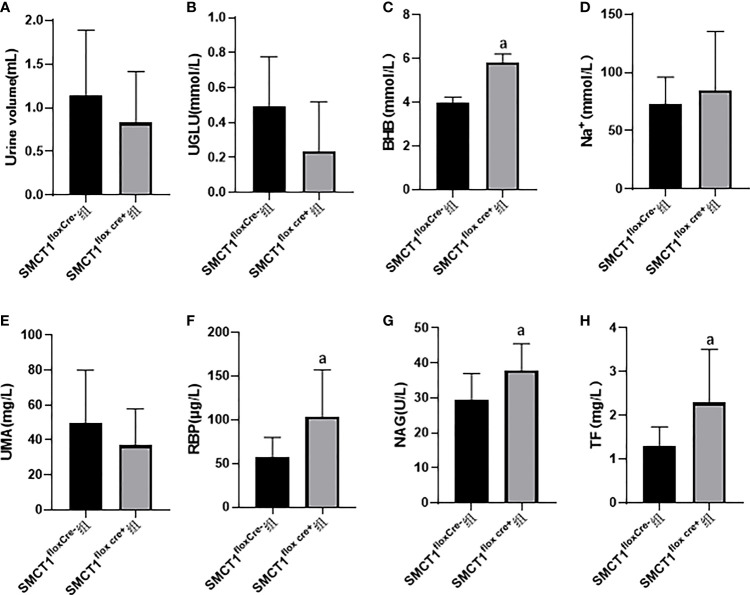
Roles of SMCT1 in tubular protection in mice starved for 48 h. SMCT1^flox cre+^ represents SMCT1 ^flox/flox cre+^ mice with specific SMCT1 gene knockout in renal tubules, and SMCT1^flox cre-^ represents wild-type SMCT1^flox/flox cre-^ mice. The level of urine volume **(A)**, UGLU **(B)**, urine BHB **(C)**, urine Na+ **(D)** ,UMA **(E)**, RBP **(F)**, NAG **(G)**, and TF **(H)** in two groups of mice. UGLU, urine glucose; BHB, urine β-hydroxybutyrate; Na^+^, urine sodium ion; UMA, microalbuminuria; RBP, urine retinol binding protein; NAG, urine N-acetyl -β –glucosaminidase; and TF, transferrin. ^a^p < 0.05, compared with the SMCT1^flox cre-^ group.

After 48 h of starvation, there were no significant differences in the urine volume, urine glucose, and urine Na+ between the SMCT1^flox/floxCre+^ group and the SMCT1^flox/floxCre-^ group (p > 0.05) (**Figures 2A, B, D**). Compared with the SMCT1flox/flox Cre- group, the mice in the SMCT1flox/flox Cre+ group exhibited an increased level of urine BHB (p < 0.05) (**Figure 2C**); the indices of renal injury (RBP, NAG, and TF) were also increased (p < 0.05) (**Figures 2F-H**); the urine UMA level did not changed (p > 0.05) (**Figure 2E**).

### 3.3 Metabolic switch of HK2 cells to ketone body utilization under low glucose and oxygen environment

**Figure 3:**
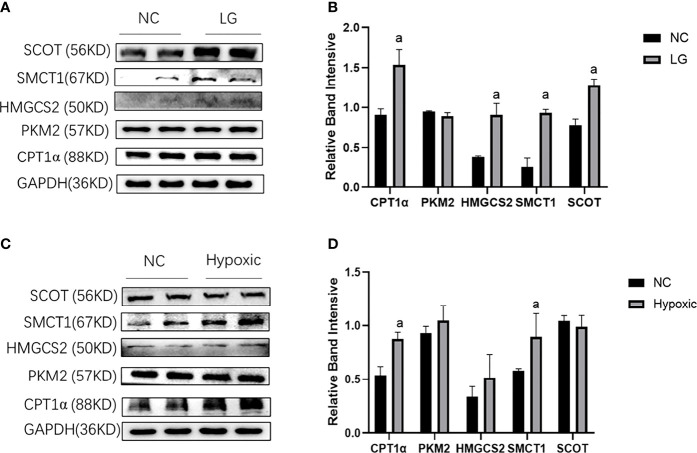
Metabolic flexibility of HK2 cells under low glucose and oxygen conditions. **(A, B)** Expression of CPT1α, PKM2, HMGCS2, SMCT1, and SCOT in HK2 cells under hypoglycemic environment; **(C, D)** Expression of CPT1α, PKM2, HMGCS2, SMCT1, and SCOT in HK2 cells under hypoxic condition; ^a^p < 0.05, compared with the NC group.

In the low-glucose group (LG group), CPT1α reflecting fatty acid oxidation, HMGCS2 reflecting ketone body synthase, SMCT1 reflecting ketone body reabsorption, and SCOT reflecting ketone body utilization were increased (p < 0.05); PKM2 reflecting glycolysis was not changed compared with the NC group (p > 0.05) (**Figures 3A, B**). In the hypoxic group, the expression of CPT1α and SMCT1 was increased in HK2 cells (p < 0.05), and the expression of PKM2, HMGCS2, and SCOT did not change compared with the NC group (p > 0.05) (**Figures 3C, D**).

The above results infer a transfer of HK2 cells to utilize fatty acid and ketone bodies under low glucose and oxygen environment, especially with respect to ketone bodies.

### 3.4 Effects of low-level BHB on the mitochondrial and cell function in HK2 cells under low glucose or hypoxic environment

#### 3.4.1 Mitochondrial function and ATP production

**Figure 4:**
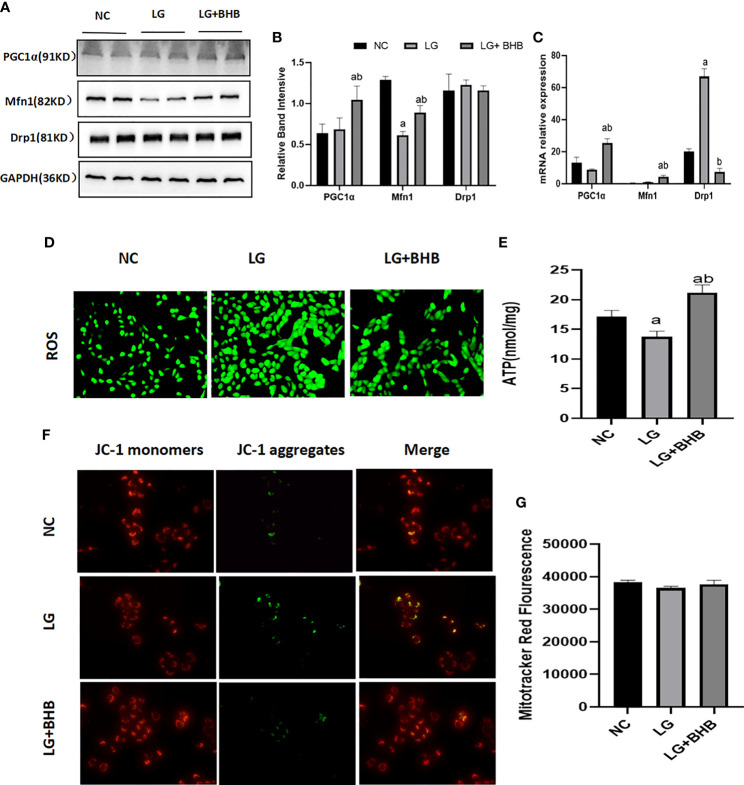
Effect of BHB on the mitochondrial function in HK2 cells under low glucose condition. **(A–C)** Expression of PGC1α, Mfn1, and Drp1 in HK2 cells with or without 2 mmol/l BHB; **(D–G)** Changes of ROS, ATP production, mitochondrial membrane potential and mitochondrial number. ^a^p < 0.05, compared with the NC group; ^b^p < 0.05, compared with the LG group.

Due to the marked changes in ketone body metabolism in hypoxic or low glucose circumference in HK2 cells, we investigated the roles of BHB addition on mitochondrial function in cells during an energy crisis. Compared with the NC group, the HK2 cells in the LG group showed a decreased level in Mfn1 expression (**Figures 4A–C**), ATP production (**Figure 4E**), and membrane potential (**Figure 4F**) (p < 0.05), and increased level of Drp1 mRNA expression (**Figure 4C**) and ROS production (**Figure 4D**) (p < 0.05). Compared with the LG group, the 2 mmol/l BHB supplement increased the level of PGC1/Mfn1 expression (**Figures 4A–C**), ATP production (**Figure 4E**), and membrane potential (**Figure 4F**) (p < 0.05), but decreased the Drp1 mRNA expression (**Figure 4C**) and ROS production (**Figure 4D**) (p < 0.05). The low glucose environment with or without BHB did not change the number of mitochondria compared with the normal control (**Figure 4G**) (p > 0.05).

**Figure 5:**
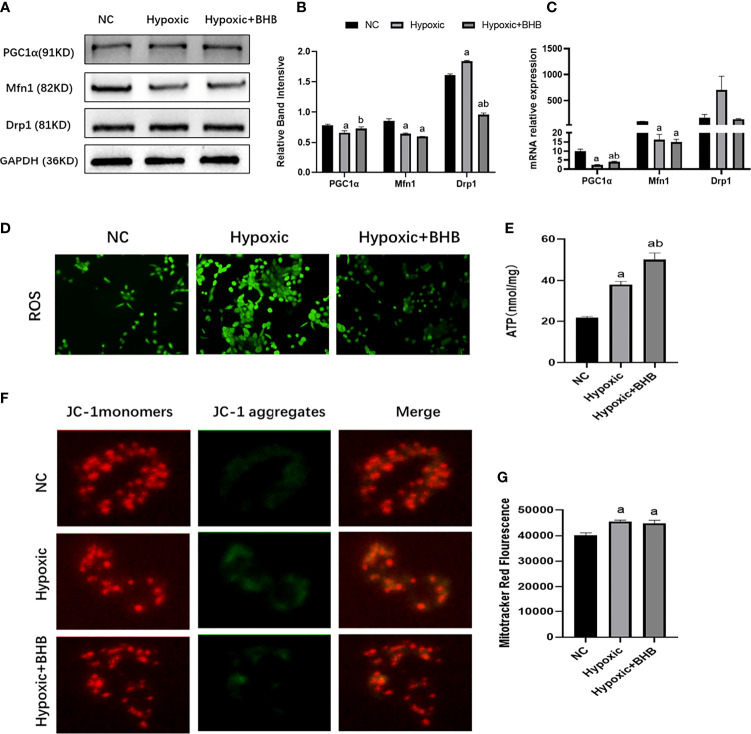
Effect of BHB supplement on the mitochondrial function in HK2 cells under hypoxic condition. **(A–C)** Expression of PGC1α, Mfn1, and Drp1 in HK2 cells under hypoxic condition with or without 2 mmol/l BHB; **(D–G)** Changes of ROS, ATP production, mitochondrial membrane potential, and the mitochondrial number, respectively. ^a^p < 0.05, compared with the NC group; ^b^p < 0.05, compared with the Hypoxic group.

Compared with the NC group, the PGC1α and Mfn1 expression (**Figures 5A–C**), ATP production (**Figure 5E**), and membrane potential (**Figure 5F**) were decreased, while Drp1 expression (**Figures 5A–C**), ROS production (**Figure 5D**), and the mitochondrial number (**Figure 5G**) increased in the hypoxic group (p < 0.05). The BHB supplement (2 mmol/l) increased the PGC1α expression, ATP production, and membrane potential (p < 0.05), and decreased the Drp1 expression and ROS production (p < 0.05). BHB did not affect the Mfn1 expression and mitochondrial number (p > 0.05).

#### 3.4.2 Albumin endocytosis and apoptosis

**Figure 6:**
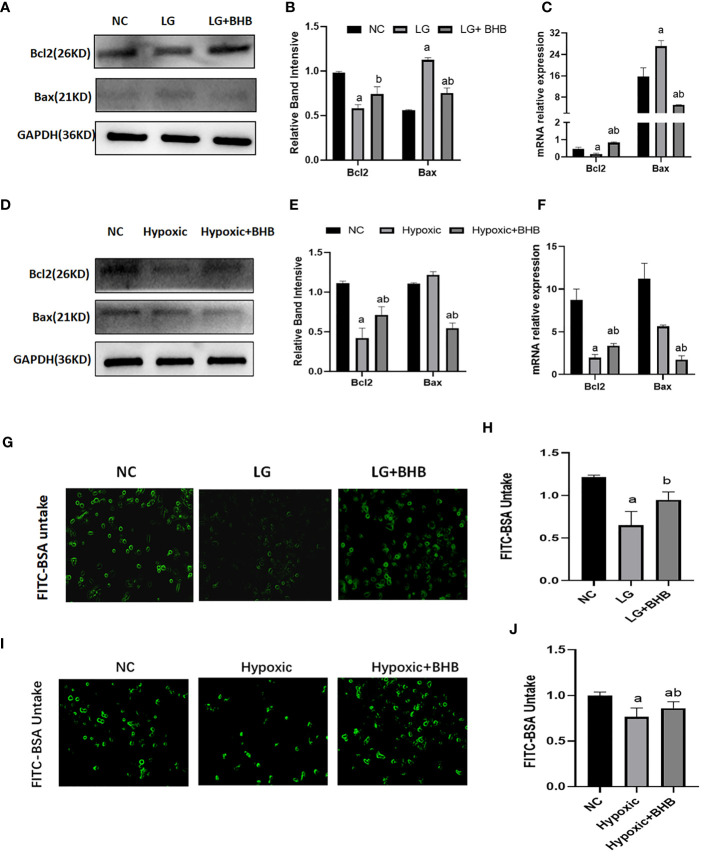
Effect of BHB on apoptosis and albumin endocytosis in HK2 cells under hypoglycemic and hypoxic conditions. **(A, D)** Expression of Bcl-2 and Bax; **(B, E)** Quantitative results of Bcl-2 and Bax proteins; **(C, F)** Quantitative results of Bcl-2 and Bax gene expression; **(G, I)** Changes of endocytic albumin in different groups detected by fluorescence microscopy; **(H, J)** Changes of endocytic albumin in different groups detected by a microplate reader. In panels **(B, C, H)** ^a^p < 0.05, compared with the NC group; ^b^p < 0.05, compared with the LG group; in panels **(E, F, J)** ^a^p < 0.05, compared with the NC group; ^b^p < 0.05, compared with the Hypoxic group.

Compared with the NC group, Bax expression increased whereas the Bcl-2 expression decreased both in the LG and Hypoxic groups (p < 0.05) (**Figures 6A–F**); albumin endocytosis decreased in the LG and Hypoxic groups (p < 0.05) (**Figures 6G–J**). The addition of 2 mmol/l BHB improved Bcl-2 expression (p < 0.05), Bax expression, and albumin endocytosis (p < 0.05).

### 3.5 High insulin inhibits the metabolic switch to BHB in HK2 cells under low-glucose or hypoxic situation

#### 3.5.1 Metabolic switch to BHB utilization

**Figure 7:**
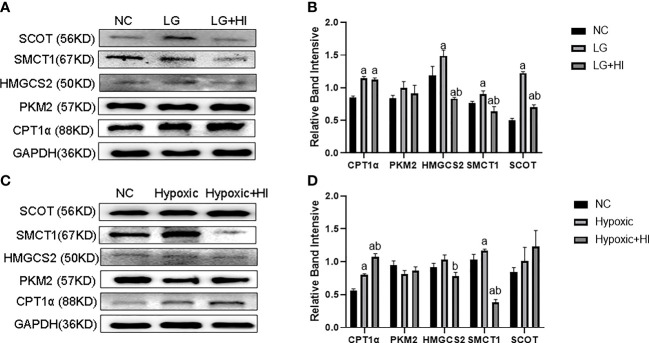
Effect of high insulin level on metabolic switch to BHB in HK2 cells under hypoglycemic and hypoxic conditions. **(A)** Expression of CPT1α, PKM2, HMGCS2, SMCT1, and SCOT in HK2 cells under high insulin and low glucose conditions; **(B)** Quantitative results of corresponding proteins expression in panels **(A)**; **(C)** Expression of CPT1α, PKM2, HMGCS2, SMCT1, and SCOT in HK2 cells under high insulin and low oxygen conditions; **(D)** Quantitative results of the corresponding proteins expression in panel **(C)**. ^a^p < 0.05, compared with the NC group; ^b^p < 0.05, compared with the LG/Hypoxic group.

Compared with the LG group, the high insulin level decreased HMGCS2, SMCT1, and SCOT expression (p < 0.05), while it did not change the CPT1α and PKM2 expression in HK2 cells under low glucose condition (**Figures 7A, B**) (p > 0.05). Compared with the Hypoxic group, the high insulin level increased the CPT1α expression and decreased the expression of HMGCS2 and SMCT1, but did not affect the expression of PKM2 and SCOT (**Figures 7C, D**) (p < 0.05).

#### 3.5.2 Mitochondrial function and ATP production

**Figure 8:**
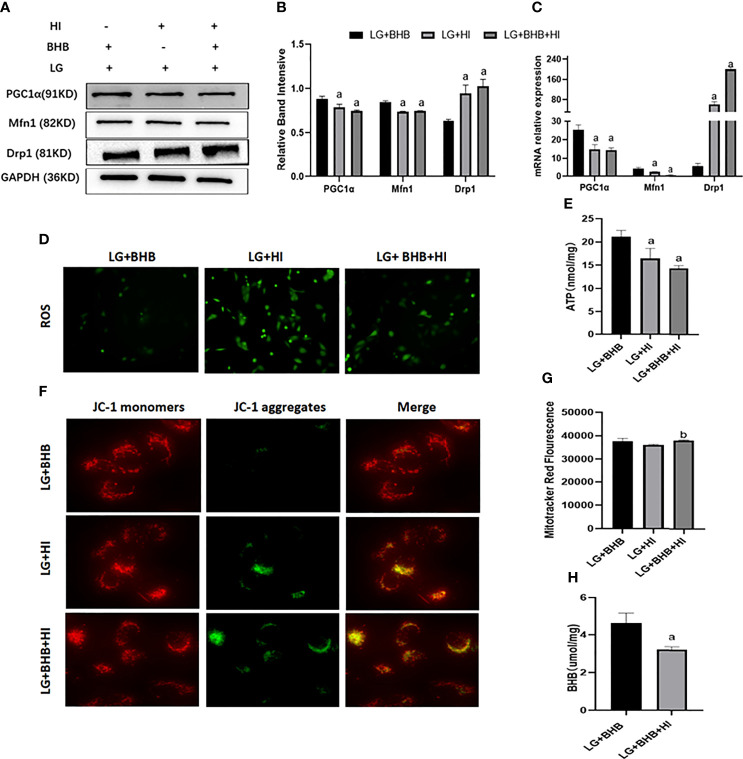
Effect of high insulin on BHB induced protection of mitochondria in HK2 cells cultured under low glucose culture. **(A)** Expression of PGC1α, Mfn1, and Drp1; **(B, C)** Quantitative results of protein and gene expression of PGC1α, Mfn1, and Drp1, respectively; **(D–H)** Changes of ROS, ATP production, mitochondrial membrane potential, the mitochondrial number changes, and intracellular BHB content. ^a^p < 0.05, compared with the LG+BHB group; ^b^p < 0.05, compared with the LG+HI group.

Compared with the LG+BHB group, the high insulin level decreased the expression of PGC1α and Mfn1 (p < 0.05), while increasing the expression of Drp1 (p < 0.05) in HK2 cells (**Figures 8A–C**). Further, the high insulin level increased the ROS production (**Figure 8D**), but decreased the ATP production, mitochondrial membrane potential, and intracellular BHB content (**Figures 8E–H**) (p < 0.05). The high insulin level did not change the mitochondrial number (p > 0.05) (**Figure 8G**). Therefore, high insulin levels may inhibit the mitochondrial function by inhibiting the BHB uptake.

**Figure 9:**
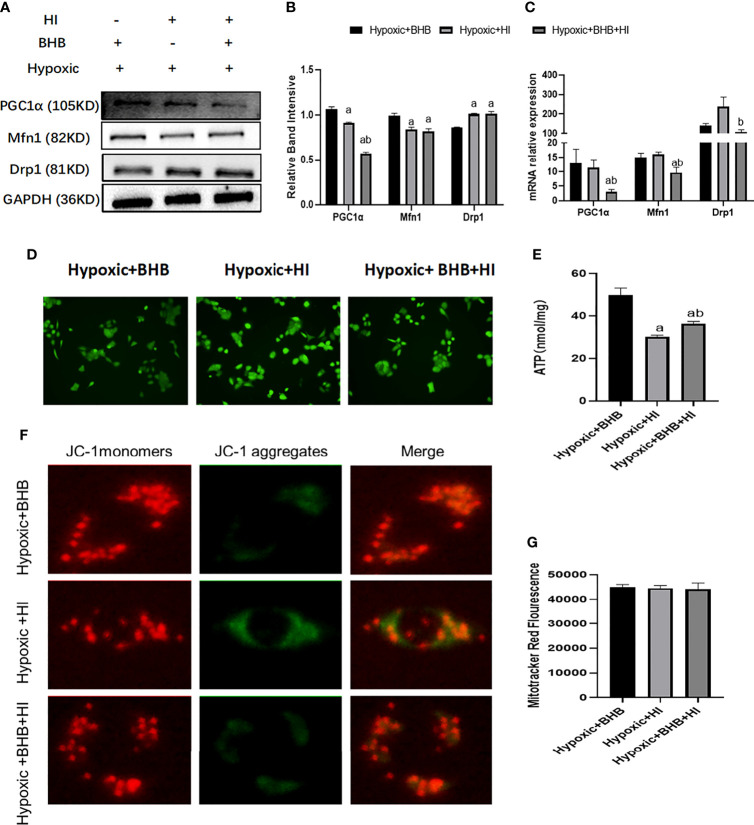
Effect of high insulin on BHB induced protection of mitochondrial in HK2 cells cultured in hypoxic environment. **(A)** Expression of PGC1α, Mfn1, and Drp1; **(B, C)** Quantitative results of protein and gene expression of PGC1α, Mfn1, and Drp1, respectively; **(D–G)** Changes of ROS, ATP production, mitochondrial membrane potential, and the mitochondrial number. ^a^p < 0.05 compared with the Hypoxic + BHB group; ^b^p < 0.05, compared with the Hypoxic + HI group.

Compared with the Hypoxic + BHB group, HK2 cells in the Hypoxic + BHB + HI group exhibited decreased PGC1α/Mfn1 expression (**Figures 9A–C**), ATP production (**Figure 9E**), and mitochondrial membrane potential (**Figure 9F**) (p < 0.05), but increased Drp1 expression (**Figures 9A, B**) and ROS production (**Figure 9D**), while the mitochondrial number was not affected (**Figure 9G**) (p > 0.05). In general, the protective effect of BHB on the mitochondrial function in HK2 cells in the hypoxic environment was also inhibited by the high insulin level.

#### 3.5.3 Albumin endocytosis and apoptosis

**Figure 10:**
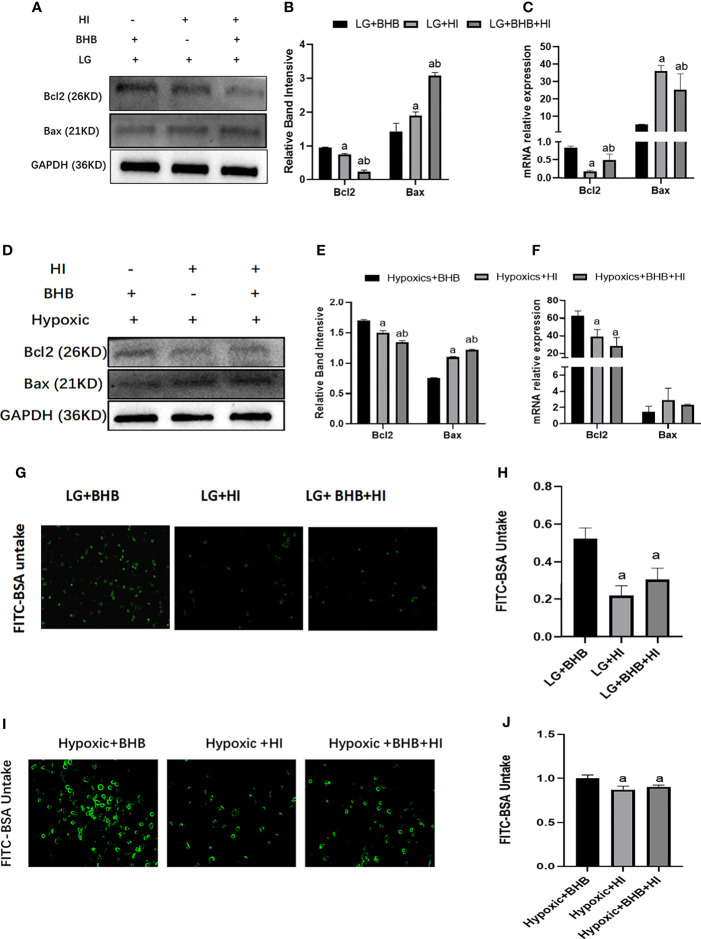
Effect of high insulin on BHB induced protection on apoptosis and endocytic albumin in HK2 cells under low glucose or hypoxic conditions. **(A, D)** Expression of Bcl-2 and Bax; **(B, E)** Quantitative results of Bcl-2 and Bax proteins; **(C, F)** Quantitative results of Bcl-2 and Bax gene expression; **(G, I)** Changes of endocytic albumin detected by fluorescence microscopy; **(H, J)** Changes of endocytic albumin detected by a microplate reader. In panels **(B, C, H)** ^a^p < 0.05, compared with the LG+BHB group; ^b^p < 0.05, compared with the LG+HI group; in panels **(E, F, J)** ^a^p < 0.05, compared with the Hypoxic + BHB group; ^b^p < 0.05, compared with the Hypoxic + HI group.

High insulin reversed the expression of Bcl-2 expression (p < 0.05), Bax expression (p < 0.05) (**Figures 10A–F**), and albumin endocytosis (p < 0.05) (**Figures 10G–J**) induced by BHB supplement under low glucose or hypoxic condition. These results suggest that a high insulin level inhibits the protection of BHB on apoptosis and endocytosis albumin function during an energy crisis.

### 3.6 High insulin affects BHB reabsorption for ATP production *via* SIRT3/SMCT1 pathway

**Figure 11:**
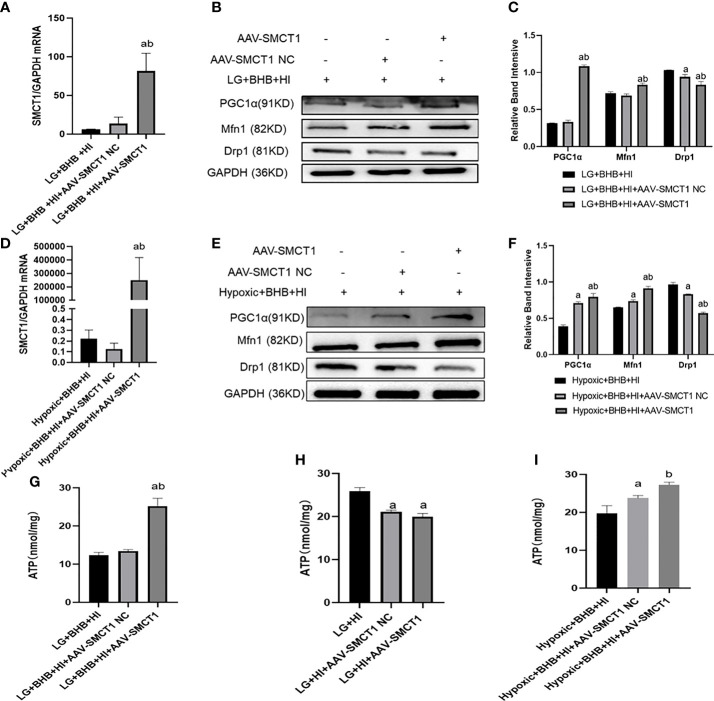
High insulin level inhibited BHB-induced protection on mitochondrial function in HK2 cells under low glucose or hypoxic conditions through SMCT1 in HK2 cells. **(A, D)** Validation of overexpression of SMCT1; **(B, C, E, F)** Expression of PGC1α, Mfn1, and Drp1 in HK2 cells under overexpression of SMCT1; **(G–I)** ATP production in different groups. In panels **(A, C, G, H)** ^a^p < 0.05, compared with the LG + BHB + HI group (control group); ^b^p < 0.05, compared with the LG + BHB + HI + AAV-SMCT1 NC group (vector group); in panels **(D, F, I)** ^a^p < 0.05, compared with Hypoxic + BHB + HI group; ^b^p < 0.05, compared with the Hypoxic +BHB + HI + AAV-SMCT1 NC group.

Due to the decreased expression of SMCT1 in cells treated with high insulin, we hypothesized that high insulin may decrease the cellular ability of metabolic switch to BHB for energy supply *via* inhibition of SMCT1 expression under low glucose or hypoxic condition. In addition to the high expression of SMCT1 (AAV-SMCT1) (**Figures 11A, D**), the level of PGC1/Mfn1 expression increased, but the level of Drp1 expression decreased (**Figures 11B, C, E, F**); intracellular ATP production increased in cells supplied with BHB under low glucose or hypoxic condition (p < 0.05) (**Figures 11G, I**). However, ATP did not increase in cells without BHB in culture medium (**Figure 11H**) under low glucose or hypoxic condition, implying that SMCT1 plays an important role by absorbing BHB, and overexpression of SMCT1 can improve the mitochondrial function and cellular ATP production of HK2 cells.

**Figure 12:**
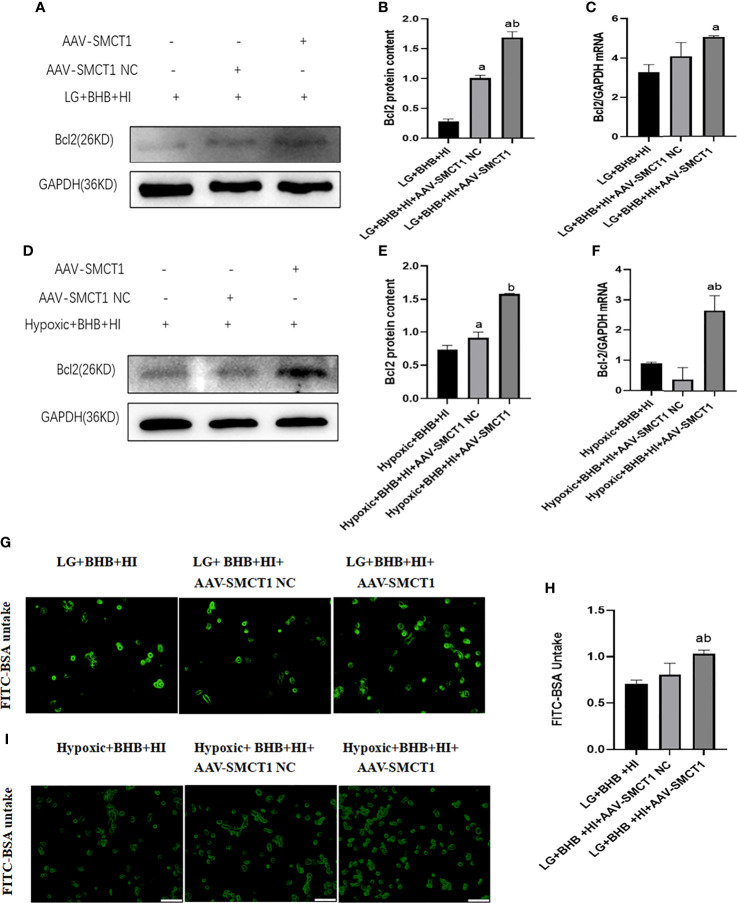
High insulin level inhibits BHB-induced protection on apoptosis and endocytic albumin production in HK2 cells under low glucose or hypoxic condition through SMCT1 in HK2 cells. **(A, D)**, Expression of Bcl-2; **(B, E)** Quantitative results of Bcl-2 proteins; **(C, F)** Quantitative results of Bcl-2 gene expression; **(G, I)** Changes of endocytosed albumin detected by fluorescence microscopy; **(H)** Changes of endocytosed albumin detected by a microplate reader. In panels **(B, C, H)** ^a^p < 0.05 compared with the LG+BHB+HI group; ^b^ p < 0.05, compared with the LG + BHB + HI + AAV-SMCT1 NC group; in panels **(C, F)** ^a^p < 0.05, compared with the Hypoxic + BHB + HI group; ^b^p < 0.05, compared with the Hypoxic + BHB + HI + AAV-SMCT1 NC group.

Upon overexpression of SMCT1, the level of Bcl2 expression (**Figures 12A–F**) and albumin endocytosis (**Figures 12G-I**) increased under high insulin level in HK2 cells (p < 0.05). In summary, the inhibition of high insulin on BHB utilization may partly depend on its inhibition on SMCT1 expression in proximal renal tubular epithelial cells under low glucose or hypoxic environments.

**Figure 13:**
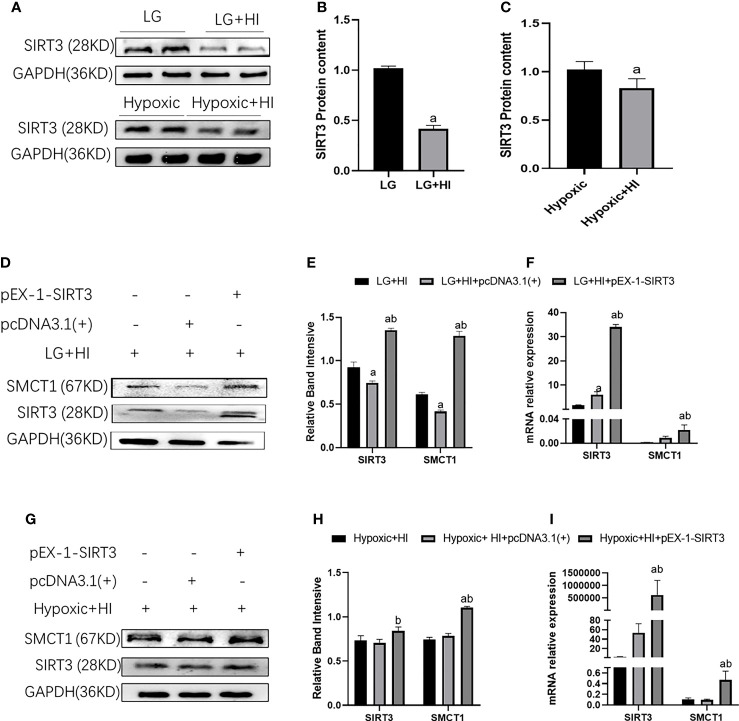
High insulin inhibits SMCT1 expression by suppressing SIRT3 expression. **(A)** Expression of SIRT3 expression; **(B)** Quantitative results of the corresponding protein expression in **(A)**; **(C)** Quantitative results of SIRT3 mRNA expression. **(D, G)** Expression of SIRT3 and SMCT1; **(E, H)** Quantitative results of SIRT3 and SMCT1 expression; **(E, H)** Quantitative results of SIRT3 and SMCT1 mRNA expression. In panels **(B)** ^a^p < 0.05, compared with the LG group, in Figure **(C)** ^a^p < 0.05, compared with the Hypoxic group, in panels **(E, F)** ^a^p < 0.05, compared with the LG + HI group; ^b^p < 0.05 compared with the LG + HI + pcDNA3.1(+) group; in panels **(H, I)** ^a^p < 0.05, compared with the Hypoxic + HI group; ^b^p < 0.05, compared with the Hypoxic + HI + pcDNA3.1(+) group.

In our previous study, we reported that a high insulin level inhibits the expression of SIRT3 (8). Therefore, high insulin levels may inhibit the expression of SMCT1 *via* inhibition of SIRT3. As expected, a high insulin level inhibited the expression of SIRT3 protein and mRNA (p < 0.05) (**Figures 13A–C**). The over-expression of SIRT3 upregulated the expression of SMCT1 (p < 0.05) (**Figures 13D–I**) as well as ATP production and mitochondrial function **Table 2**.

## 4 Discussion

An increasing number of studies have reported that caloric restriction and hypoxia can induce metabolic changes in various organs, including the heart, liver, kidney, and brain to resist cell damage caused by an energy crisis (10–13). This ability of cells to shift energy metabolic pathways in energy crises is metabolic flexibility; however, the exact mechanism of metabolic flexibility remains unclear (3, 14). Our results indicated that the expression of CPT1α (a key enzyme in fatty acid oxidation), and key enzymes of ketone body metabolism (HMGCS2, SMCT1, and SCOT) was increased in HK2 cells under low glucose and oxygen conditions. Concomitantly, no significant changes were observed in PKM2, a key enzyme in glycolysis. The above results infer an MF of HK2 cells to utilize fatty acid and ketone bodies under low glucose and oxygen conditions, especially for ketone bodies, which is an efficient fuel. The fuel efficiency is measured by the P/O ratio, which is the number of ATP molecules (P) produced per oxygen atom (O) in the mitochondrial electron transport chain. The P/O ratio for glucose, fatty acids, and ketones is 2.58, 2.33, and 2.50, respectively (15–17). Although the oxidation of fatty acids produces more ATP (106 ATP), which favors renal tubules with a high metabolic demand, it requires a higher oxygen consumption and produces excess reactive oxidants (ROS), which is unfavorable to tissues in hypoxia. For every two carbons, ketone bodies produce more ATP than fatty acids and produce less ROS than glucose (18). Therefore, increased ketone body utilization is a manifestation of MF in proximal renal tubular epithelial cells in a starved and hypoxic environment.

Mitochondria, the main sites of cellular energy production, participate in the regulation of cell MF and are related to protein synthesis, redox, and apoptosis, among others. Mitochondrial damage can lead to reduced MF and the release of apoptosis-related factors, leading to an impaired cell structure and function, and even apoptosis (19–21). Subsequently, whether BHB protects HK2 cells through the improvement of mitochondrial energy production and function remains unclear. To this end, we further investigated the effects of BHB, as an energy substrate, on mitochondrial function-related proteins, ATP production, ROS production, membrane potential, and the mitochondrial number under low glucose and oxygen conditions. Fusion and fission are two processes necessary to maintain the mitochondrial function: fusion elongates mitochondria and helps oxidative phosphorylation to maintain ATP production and redistribute mitochondrial proteins; fission separates damaged mitochondria, and if damaged mitochondria cannot be recycled, cytochrome c (Cyt-c), released into the cytoplasm, will increase significantly and induce apoptosis (22). In diabetic rats, increased mitochondrial fission was found to be associated with renal injury, including impaired renal function (23), while Mfn1 reflects on mitochondrial fusion and mitochondrial fission is reflected on Drp1. PGC1α is a mitochondrial biosynthesis-related protein that reflects on new mitochondrial formation; additionally, mitochondrial biosynthesis contributes to the production of new functional mitochondria that can adapt to an increased energy demand (19). Our results show that BHB promotes mitochondrial ATP production, expression of PGC1α, Mfn1 mRNA, and proteins, and decreases the expression of Drp1 mRNA in HK2 cells under a starved environment, which reflects the increased energy demand under starvation. BHB can not only promote the production of PGC1α to generate new functional mitochondria, thus increasing ATP. Notably, BHB can promote mitochondrial fusion and increase oxidative phosphorylation, which further increases ATP production. Moreover, a reduction in the expression of Drp1 could prevent cellular damage from impaired mitochondria, resulting in decreased ROS production and the restoration of mitochondrial membrane potential. In contrast, in the hypoglycemic environment, cellular ATP was not reduced in the hypoxic environment, possibly because the normal glucose concentration of the medium used in the hypoxic environment increased the use of glucose in proximal renal tubular epithelial cells, and the addition of BHB further increased cellular ATP production. Moreover, BHB did not increase the Mfn1 protein and mRNA expression in a hypoxic environment. In conclusion, BHB has beneficial effects on energy production and mitochondrial function in starved or hypoxic environments. Our findings are consistent with the results of other studies, such as Ahola-Erkkila et al. (24) Their survey of a mitochondrial myopathy model found that BHB helped maintain mitochondrial respiration and morphology within the muscle tissue. Parker et al. (25) also found that 5 mmol/l BHB promoted the proliferative viability of muscle cells, while the cellular mitochondrial function was significantly improved. Thus, our study supports that BHB is a useful supplemental energy source for renal tubular epithelial cells in a starved, hypoxic environment, improving ATP production and mitochondrial function, promoting cell survival. Any factor that affects the BHB energy supply may lead to cellular dysfunction and promote the development of urinary protein.

Hyperinsulinemia is common in patients with type 2 diabetes, which may occur at a time-point even years prior to the onset of diabetes. At this time, the blood glucose of the patient is normal or even lower than normal because of the delayed peak insulin secretion (26–28). Besides, the uptake of glucose for HK2 cells in insulin resistance may be weakened because of the impairment of the phosphoinositide 3-kinase (PI3K)/AKT signaling pathway, or perturbations in the trafficking of glucose transporters (GLUTs), which mediate the uptake of glucose into the cells (29). Our previous study found that in the presence of high-concentration insulin, the IRS-1/PI3-K/Akt signaling pathway in the renal tubular epithelial cells was inhibited (30). In addition, several studies have confirmed that the oxygen level is lower in the tubular epithelium in animal models of obesity, IGT, or hyperlipidemia, etc. (31–34). Therefore, the tubular epithelial cells are short of energy because of the relatively low glucose and oxygen levels in the kidney. At this time, the ketone bodies may become an effective supplementary energy source for cell energy supply. A growing body of research suggests that exogenous ketones protect from acute kidney injury and chronic kidney disease. β-Hydroxybutyrate can decrease kidney ischemia and reperfusion injury by activating FOXO3 and inhibiting pyroptosis (35), while a 1,3-butanediol-rich (a precursor to BHB) diet diminishes macrophage infiltration, interstitial fibrosis, apoptosis, and albuminuria, subsequently reversing diabetic nephropathy (36). The improved kidney outcome in patients with SGLT2 inhibitors can be explained at least in part by its ketogenic effects (36–40). A previous study has shown the kidney BHB level to be low in obese rats; however, the mechanism remains unclear (9). Our study found that a high insulin secretion inhibited ketone metabolism and decreased the intracellular BHB level, which led to decreased ATP production, mitochondrial biosynthesis, and increased ROS production and cell apoptosis. However, addition of 2 mmol/l of BHB counteracted the alterations induced by high insulin, decreased cell apoptosis, and improved cell function. Thus, our study confirms that hyperinsulinemia affects BHB usage in renal tubular epithelial cells under an environment of starvation and hypoxia, which may lead to mitochondrial damage and renal injury because of the energy crisis.

Our further studies investigate the mechanism by which hyperinsulinemia affects the utilization of ketone bodies in epithelial cells. Different from other cells, renal tubular epithelial cells can not only synthesize ketone bodies but also reabsorb ketone bodies from the tubular lumen of the kidney. SMCT1 is the key protein of BHB reabsorption in the kidney. A study found that high insulin level inhibited SMCT1 transport activity in African clawed toad oocytes (41), while another study confirmed that insulin decreased SMCT1 gene expression in the mouse colon tissue (42). Our results revealed that the high-fat diet decreased the expression of SMCT1 and increased the expression of HMGCS2, and the expression of SCOT remained unchanged in ZDF rats. Under diabetic conditions, the kidney exhibits abnormal energy metabolism, including impaired glucose oxidative phosphorylation and fatty acid oxidation, which causes an impaired ATP supply. The process of glycolysis is elevated; however, it fails to be compensated because of its low efficiency. We speculate that increased ketone production may be an adaptive manifestation as it is a more efficient ATP supply (43–45). Our *in vitro* studies found that high insulin levels inhibited the expression of SMCT1 in HK2 cells, while upregulating SMCT1 expression reversed the damages induced by high insulin levels in HK2 cells. Kidney damages also occurred in kidney-specific SMCT1 knockout mice starved for 48 h. Therefore, the downregulation of SMCT1 may be the mediator of renal injuries caused by hyperinsulinemia. Although a ketogenic diet is beneficial for the kidney in diabetes, polycystic kidney, and acute kidney ischemia (46–48), a ketogenic diet is not routinely recommended for the treatment of nephropathy considering long-term safety. Therefore, SMCT1 may be a new target for the prevention and treatment of kidney injury in patients with diabetic nephropathy, obesity-related nephropathy, and sleep apnea.

SIRT3 is a key regulator of energy metabolism. It regulates the mitochondrial function by targeting metabolic enzymes/proteins and participates in the regulation of cellular MF. The knockout of SIRT3 in skeletal muscle cells *in vivo* and *in vitro* leads to a shift of substrate utilization from carbohydrate oxidation to lactic acid and fatty acids, even in the feeding state, resulting in the loss of MF in skeletal muscles (49). SIRT3 can deacetylate various enzymes that regulate ketone body metabolism (50). Our study found that a high insulin level could inhibit the expression of SMCT1 as well as SIRT3, while SIRT3 overexpression reversed the inhibition of high insulin on SMCT1 expression, suggesting that high insulin/SIRT3 may play key roles in the regulation of MF by regulating the expression of SMCT1.

## Conclusion

Increased absorption and utilization of BHB is part of the metabolic flexibility of the renal tubular epithelial cells under starvation and hypoxic conditions, which exerts a protective effect on renal tubular epithelial cells by improving the mitochondrial function and cell survival, while hyperinsulinemia inhibits the absorption of BHB *via* inhibition of the SIRT3/SMCT1 pathway. The methods targeting the SIRT3/SMCT1 pathway may be a promising way to treat kidney diseases related to hyperinsulinemia as in cases of type 2 diabetes and obesity.

## Data availability statement

The datasets presented in this study can be found in online repositories. The names of the repository/repositories and accession number(s) can be found in the article/**Supplementary Material**.

## Ethics statement

This study was reviewed and approved by Experimental Animal Ethics Committee, Chu Hsien-I Memorial Hospital, Tianjin Medical University.

## Author contributions

JX and JY conceived the study, acquired and analyzed the data, and wrote the manuscript. BC and FZ analyzed the data and reviewed the manuscript. ZHG, XL, JW and ZAG acquired and researched the data. All authors contributed to the article and approved the submitted version.

## Funding

The study was supported by grants from Key Research and Development Program of Ministry of Science and Technology of China (2018YFC1314000), Tianjin Natural Science Foundation (17JCZDJC34700).

## Conflict of Interest

The authors declare that the research was conducted in the absence of any commercial or financial relationships that could be construed as a potential conflict of interest.

## Publisher's note

All claims expressed in this article are solely those of the authors and do not necessarily represent those of their affiliated organizations, or those of the publisher, the editors and the reviewers. Any product that may be evaluated in this article, or claim that may be made by its manufacturer, is not guaranteed or endorsed by the publisher.
