# Supplementary material for: Hyperinsulinemia impairs the metabolic switch to ketone body utilization in proximal renal tubular epithelial cells under energy crisis via the inhibition of the SIRT3/SMCT1 pathway
Source: Front Endocrinol (Lausanne). 2022 Sep 27;13:960835. doi: 10.3389/fendo.2022.960835 (PMC9551351; doi:10.3389/fendo.2022.960835)
Supplement: Supplementary file 1 [file DataSheet_1.pdf]

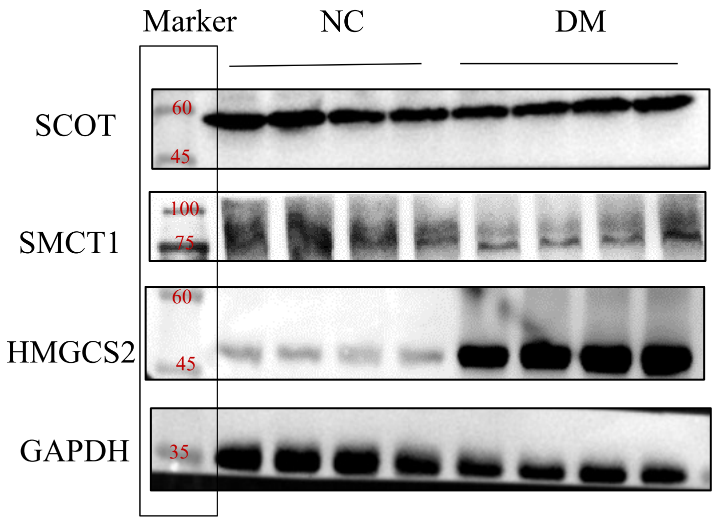
Marker
NC
DM
SCOT
60
45
SMCT1
100
75
HMGCS2
60
45
GAPDH
35

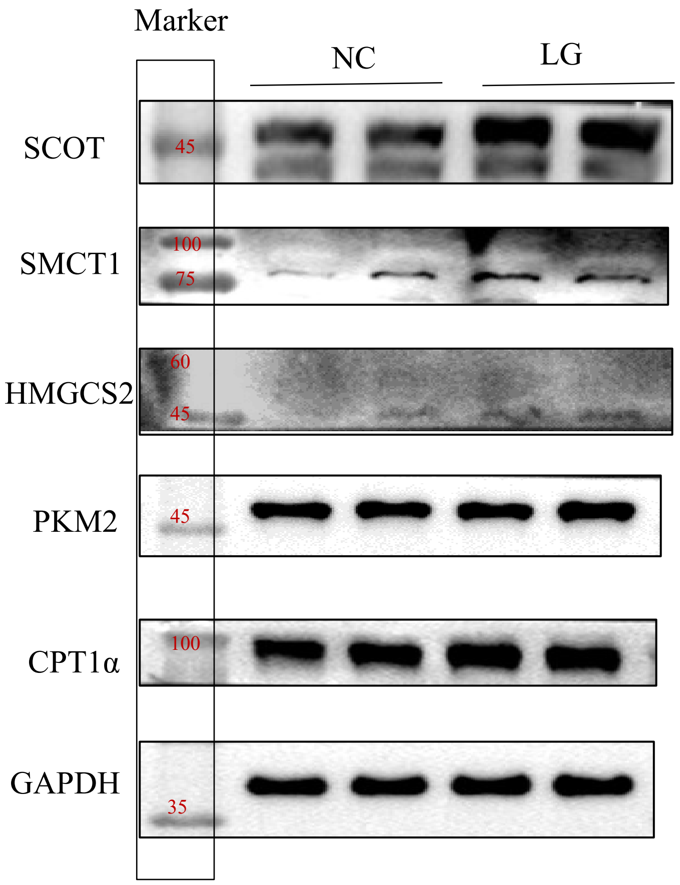
Marker
NC
LG
SCOT
45
SMCT1
100
75
HMGCS2
60
45
PKM2
45
CPT1α
100
GAPDH
35

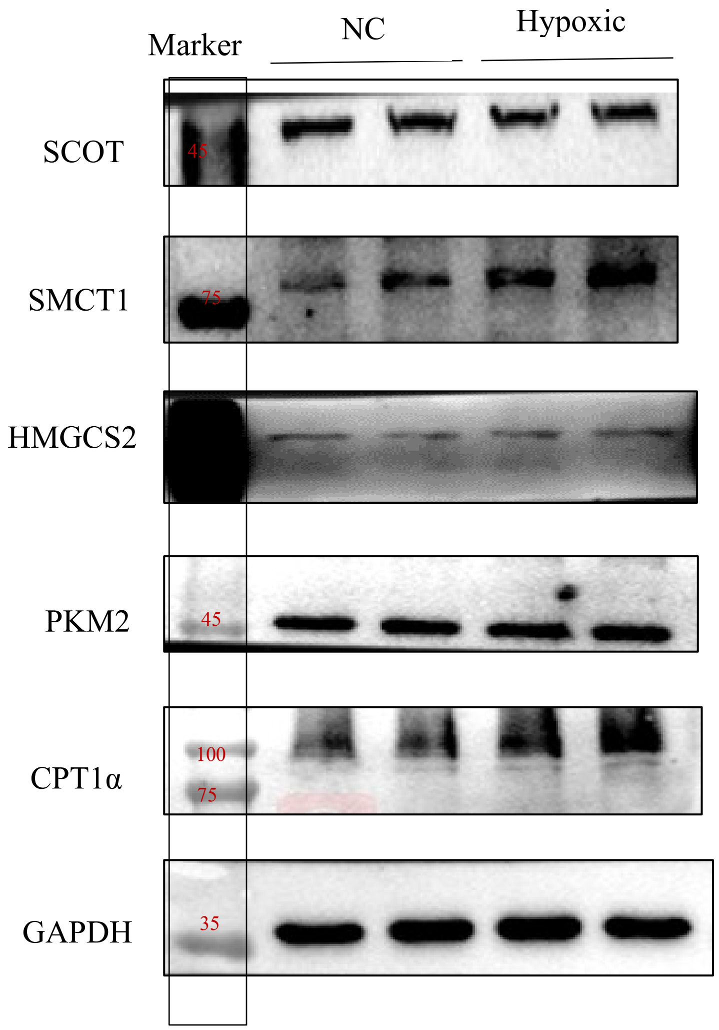
Marker
NC
Hypoxic
SCOT
45
SMCT1
75
HMGCS2
PKM2
45
CPT1α
100
75
GAPDH
35

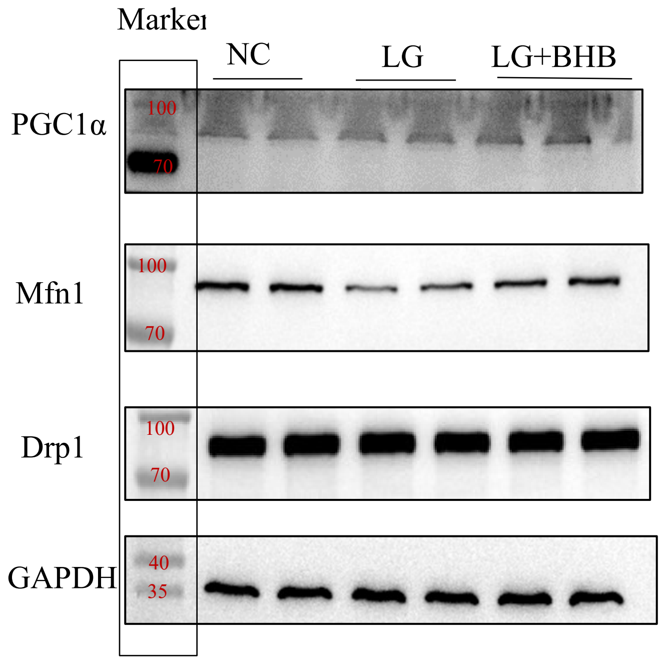
Marke
NC
LG
LG+BHB
PGC1α
100
70
Mfn1
100
70
Drp1
100
70
GAPDH
40
35

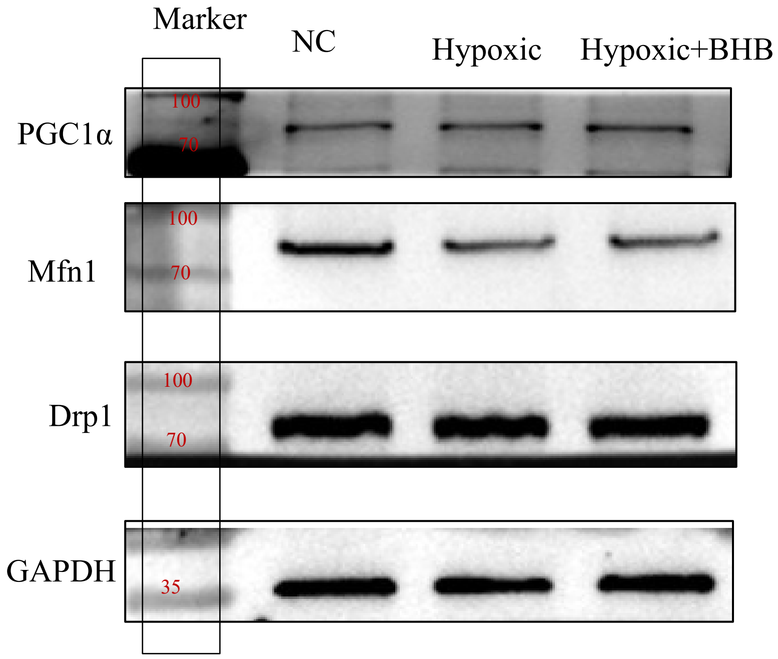
Marker
NC
Hypoxic
Hypoxic+BHB
PGC1α
Mfn1
Drp1
GAPDH
100
70
100
70
100
70
35

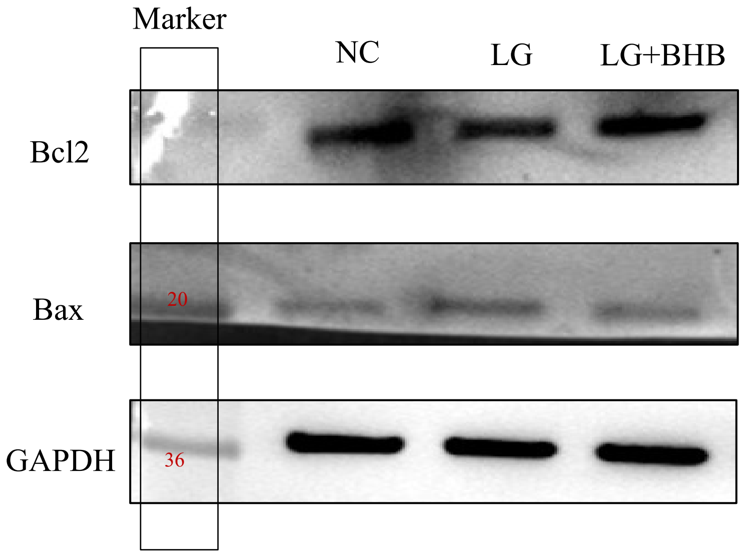
Marker
NC
LG
LG+BHB
Bcl2
Bax
GAPDH
20
36

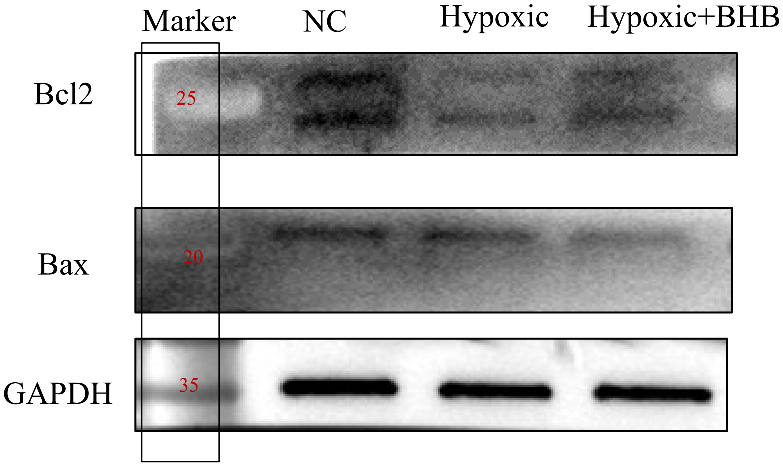
Marker
NC
Hypoxic
Hypoxic+BHB
Bcl2
Bax
GAPDH
25
20
35

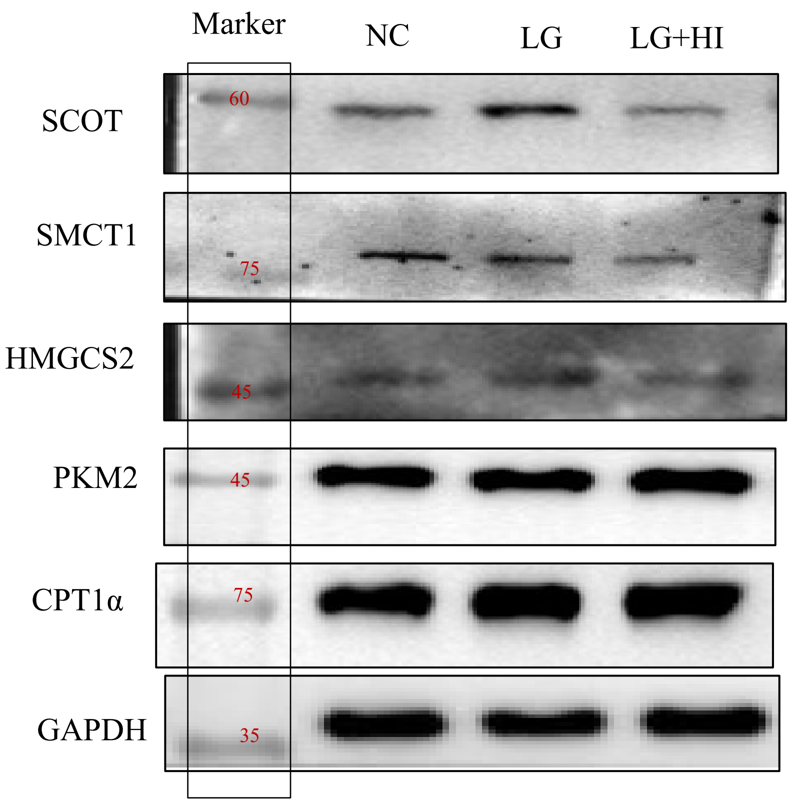
Marker
NC
LG
LG+HI
SCOT
60
SMCT1
75
HMGCS2
45
PKM2
45
CPT1α
75
GAPDH
35

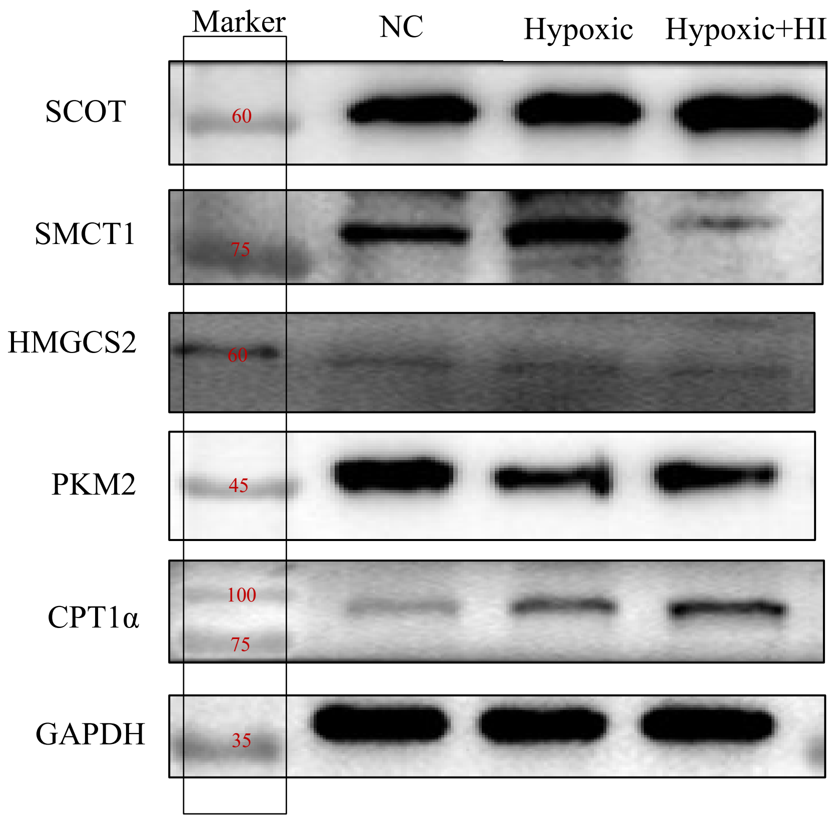
Marker
NC
Hypoxic
Hypoxic+HI
SCOT
60
SMCT1
75
HMGCS2
60
PKM2
45
CPT1α
100
75
GAPDH
35

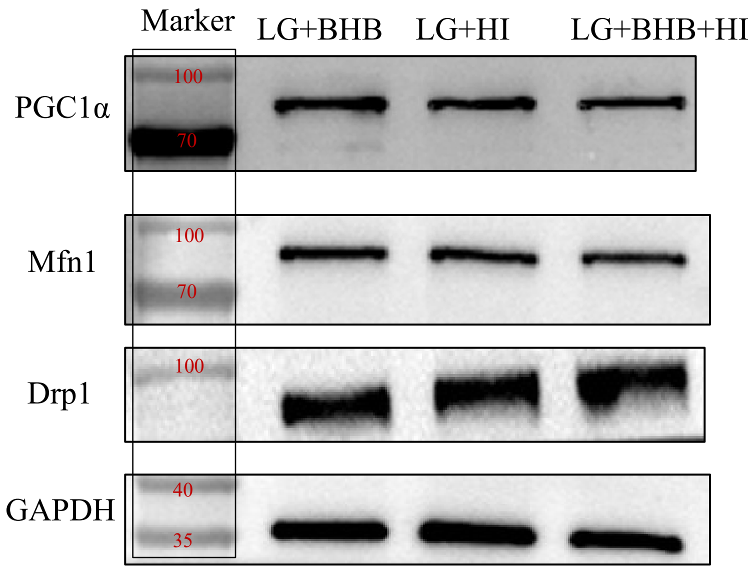
Marker
LG+BHB
LG+HI
LG+BHB+HI
PGC1α
100
70
Mfn1
100
70
Drp1
100
GAPDH
40
35

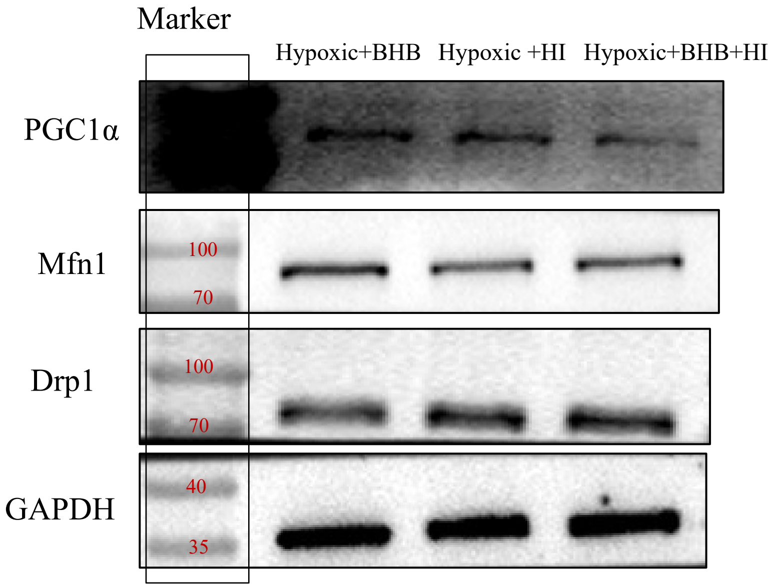
Marker
Hypoxic+BHB
Hypoxic +HI
Hypoxic+BHB+HI
PGC1α
Mfn1
100
70
Drp1
100
70
GAPDH
40
35

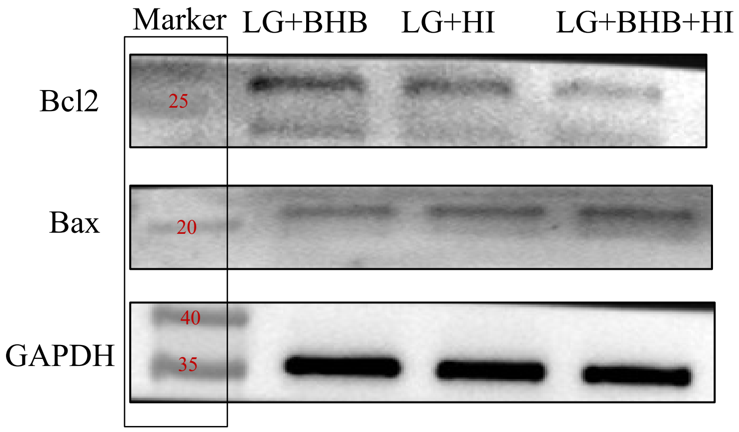
Marker
LG+BHB
LG+HI
LG+BHB+HI
Bcl2
25
Bax
20
GAPDH
40
35

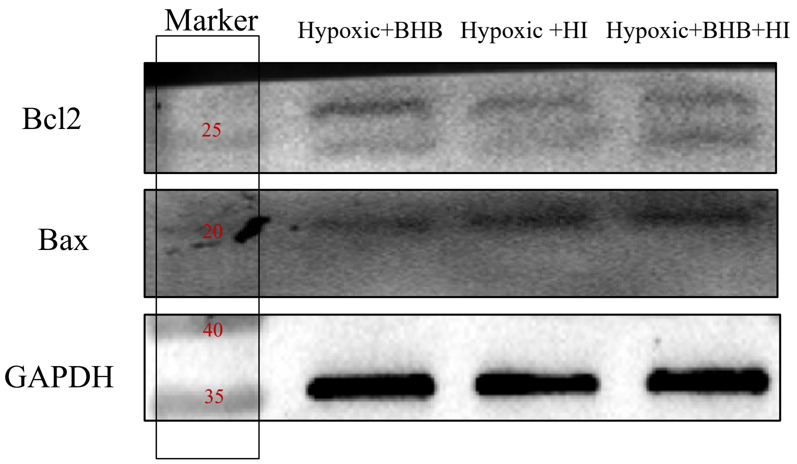
Marker
Hypoxic+BHB
Hypoxic +HI
Hypoxic+BHB+HI
Bcl2
25
Bax
20
GAPDH
40
35

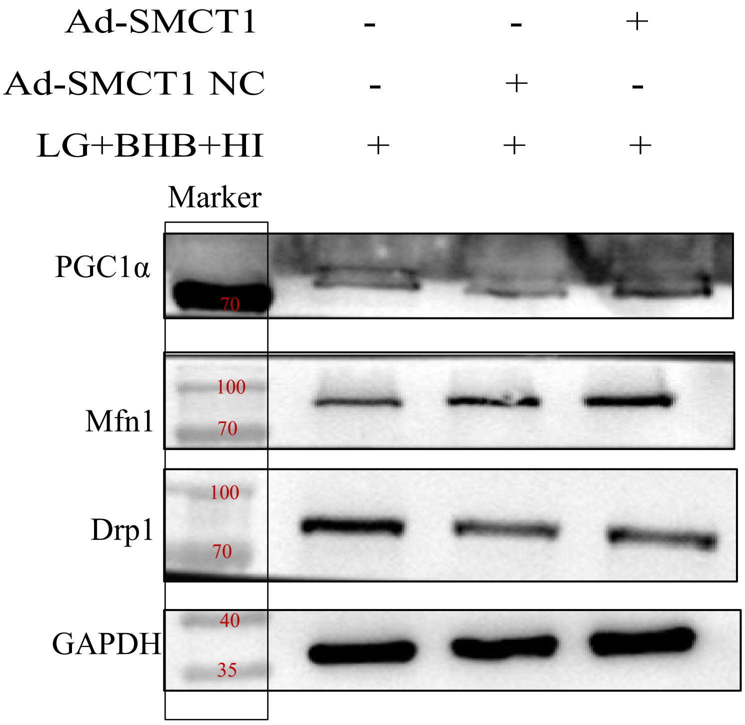
Ad-SMCT1 - - +
Ad-SMCT1 NC - + -
LG+BHB+HI + + +
Marker
PGC1α
70
Mfn1
100
70
Drp1
100
70
GAPDH
40
35

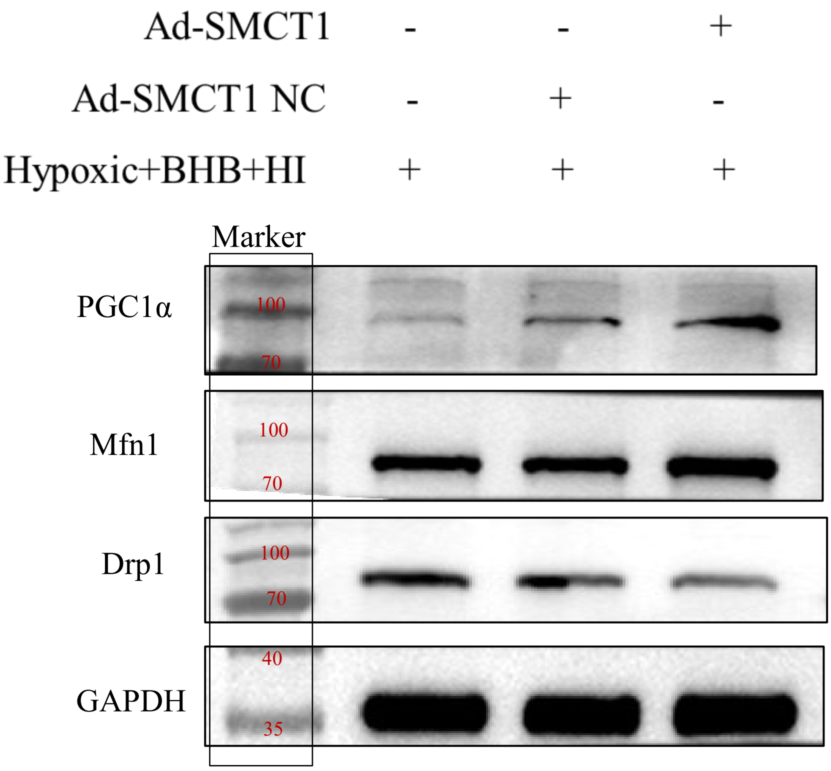
Ad-SMCT1 - - +
Ad-SMCT1 NC - + -
Hypoxic+BHB+HI + + +
Marker
PGC1α
100
70
Mfn1
100
70
Drp1
100
70
GAPDH
40
35

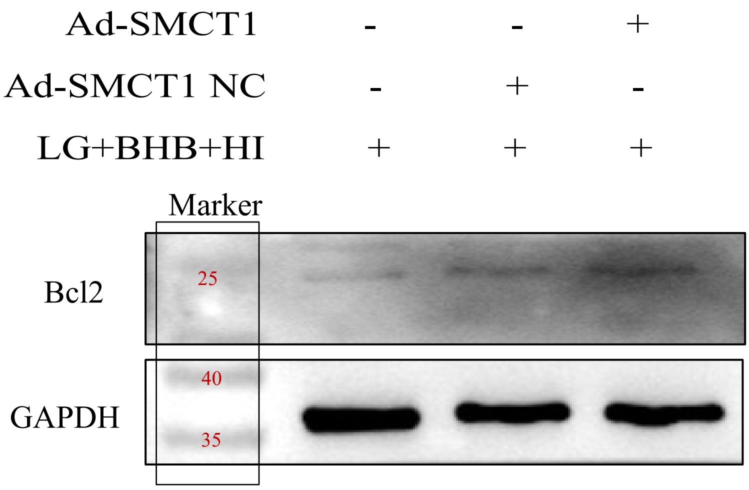
Ad-SMCT1 - - +
Ad-SMCT1 NC - + -
LG+BHB+HI + + +
Marker
Bcl2
25
GAPDH
40
35

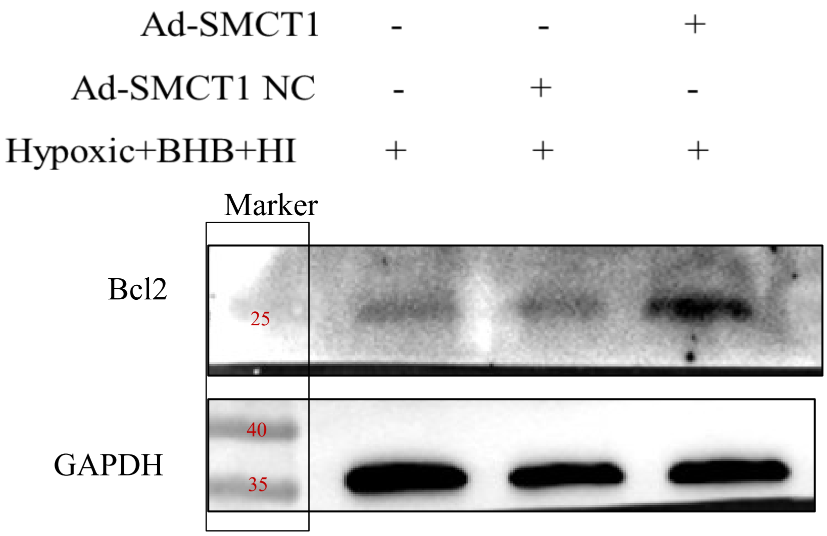

Ad-SMCT1 - - +
Ad-SMCT1 NC - + -
Hypoxic+BHB+HI + + +
Marker
Bcl2
25
GAPDH
40
35

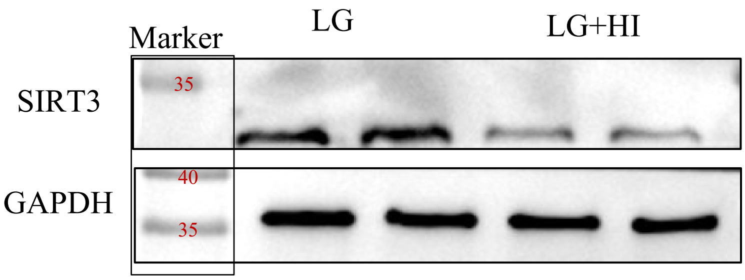

Marker
LG
LG+HI
SIRT3
35
GAPDH
40
35

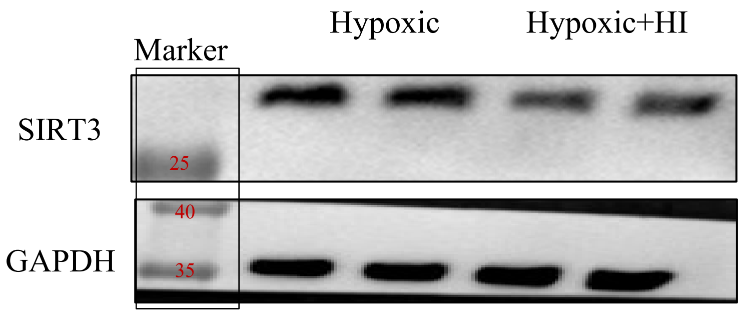

Marker
Hypoxic
Hypoxic+HI
SIRT3
25
GAPDH
40
35

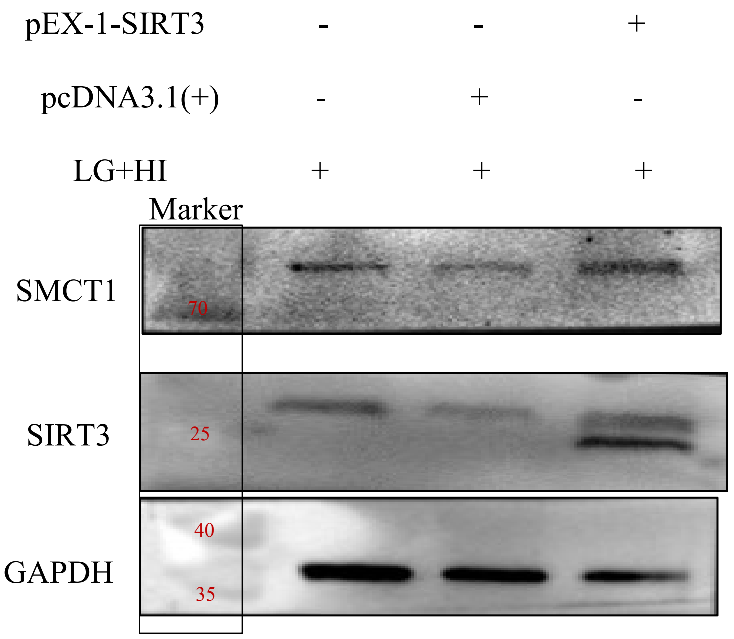
pEX-1-SIRT3 - - +
pcDNA3.1(+) - + -
LG+HI + + +
Marker
SMCT1
70
SIRT3
25
GAPDH
40
35

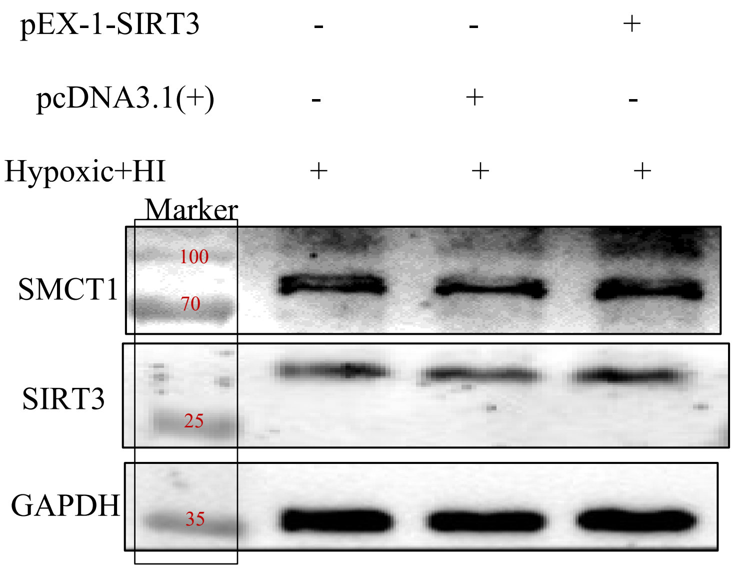
pEX-1-SIRT3 - - +
pcDNA3.1(+) - + -
Hypoxic+HI + + +
Marker
SMCT1
100
70
SIRT3
25
GAPDH
35
